# Research progress on ocular complications caused by type 2 diabetes mellitus and the function of tears and blepharons

**DOI:** 10.1515/biol-2022-0773

**Published:** 2024-01-27

**Authors:** Xiaohong Wang, Jian Fang, Lina Yang

**Affiliations:** Department of Operating Room, Xinchang County Peoples Hospital, Xinchang, 312500, Shaoxing City, Zhejiang, China; Department of Ophthalmolgy, Xinchang County Peoples Hospital, Xinchang, 312500, Shaoxing City, Zhejiang, China

**Keywords:** type 2 diabetes mellitus, ocular complications, tears, blepharons, deep learning, research progress

## Abstract

The purpose of this study was to explore the related research progress of ocular complications (OCs) caused by type 2 diabetes mellitus (T2DM), tear and tarsal function, and the application of deep learning (DL) in the diagnosis of diabetes and OCs caused by it, to provide reference for the prevention and control of OCs in T2DM patients. This study reviewed the pathogenesis and treatment of diabetes retinopathy, keratopathy, dry eye disease, glaucoma, and cataract, analyzed the relationship between OCs and tear function and tarsal function, and discussed the application value of DL in the diagnosis of diabetes and OCs. Diabetes retinopathy is related to hyperglycemia, angiogenic factors, oxidative stress, hypertension, hyperlipidemia, and other factors. The increase in water content in the corneal stroma leads to corneal relaxation, loss of transparency, and elasticity, and can lead to the occurrence of corneal lesions. Dry eye syndrome is related to abnormal stability of the tear film and imbalance in neural and immune regulation. Elevated intraocular pressure, inflammatory reactions, atrophy of the optic nerve head, and damage to optic nerve fibers are the causes of glaucoma. Cataract is a common eye disease in the elderly, which is a visual disorder caused by lens opacity. Oxidative stress is an important factor in the occurrence of cataracts. In clinical practice, blood sugar control, laser therapy, and drug therapy are used to control the above eye complications. The function of tear and tarsal plate will be affected by eye diseases. Retinopathy and dry eye disease caused by diabetes will cause dysfunction of tear and tarsal plate, which will affect the eye function of patients. Furthermore, DL can automatically diagnose and classify eye diseases, automatically analyze fundus images, and accurately diagnose diabetes retinopathy, macular degeneration, and other diseases by analyzing and processing eye images and data. The treatment of T2DM is difficult and prone to OCs, which seriously threatens the normal life of patients. The occurrence of OCs is closely related to abnormal tear and tarsal function. Based on DL, clinical diagnosis and treatment of diabetes and its OCs can be carried out, which has positive application value.

## 1 Introduction

Type 2 diabetes mellitus (T2DM) is a common metabolic disease, long-term hyperglycemia will have an impact on all organs and systems of the whole body, in which organs such as eyes, cardiovascular system, kidney, and nervous system are vulnerable to damage [1]. Ocular complications (OCs) induced by T2DM will affect ocular function and cause significant complications. OCs caused by T2DM mainly include retinopathy and non-retinopathy [2]. Diabetes retinopathy is a kind of eye disease caused by diabetes, which is one of the main causes of blindness in adults worldwide. The occurrence mechanism of diabetes retinopathy involves many factors, including hyperglycemia, hypertension, and dyslipidemia [3]. Diabetes retinopathy has a serious impact on the health of diabetes patients, and it is necessary to take preventive measures in time. In recent years, diabetes related other OCs such as corneal disease, dry eye disease, cataract, glaucoma, fundus disease, and optic neuropathy have gradually attracted the attention of doctors and diabetes patients, and it is necessary to study the pathogenesis and treatment. Therefore, this work reviewed the pathogenesis and treatment of T2DM-induced OCs, and compared and analyzed different types of diabetes and eye diseases, to offer a theoretical guidance for preventing or treating T2DM-induced OCs of patients.

Tears and blepharons are two important parts of the eyes and have active physiological functions. Tear is a transparent liquid secreted by the lacrimal gland and mainly composed of water, salts, proteins, and other components [4]. Tears lubricate the surface of the eyeball, forming a thin film over it that keeps it moist and prevents dryness and irritation. Tears also clean the eyeball and is antibacterial and antiviral to prevent eye infections while supplying oxygen and nutrients to keep the eyeball healthy [5]. Previous studies on the changes of tear secretion function in diabetes patients have been inconsistent. Andersen et al. [6] pointed out that there was no significant difference in the amount of basic tear secretion between diabetes patients and normal controls; Yeung and Dwarakanathan [7] reported that 47% of diabetes patients had decreased tear secretion. Patnaik et al. [8] showed that there was no obvious abnormality in tear film function in diabetic patients.

Blepharons are small glands in the eyelid and the skin, hair, and muscle tissue at the edge of the eyelid. Blepharons can secrete oil, lubricate tears, form a protective film to prevent excessive evaporation of tears, maintain the stability of tears, prevent the rapid evaporation of tears, and protect eyes, exerting a very important role in maintaining eye health [9]. Diabetes can lead to dry eyes, making the eyelids easy to feel dry, itchy, or painful. Patients with diabetes are more likely to suffer from blepharitis, which causes skin inflammation at the blepharitis, often accompanied by redness, itching, crusting, and other symptoms [10]. The function of tears and blepharons will be affected by eye diseases. Retinopathy and dry eye disease induced by diabetes will cause functional abnormalities of tears and blepharons, affecting the eye function of patients. Therefore, it is of important research value to analyze the correlation between T2DM-induced OCs and function of tears and blepharons.

Deep learning (DL) is widely used in the medical field and has unique application value. In recent years, DL is mainly used in clinical image analysis, clinical decision support, biomedical engineering, and health management and prevention. It has a very broad application prospect in the medical field, which can help doctors and researchers to diagnose and treat diseases more accurately, and improve the QOL and health status of patients [11,12]. Therefore, this work explored the research progress on diagnosing and treating diabetes and OCs based on DL, and analyzed the application value of DL in eye imaging image analysis. It aimed to give a guidance and reference for the prevention, control, and treatment of patients with T2DM and OCs.

## 2 Research progress of T2DM and OCs

### 2.1 Diabetic retinopathy (DR)

#### 2.1.1 Pathogenesis

**Figure 1:**
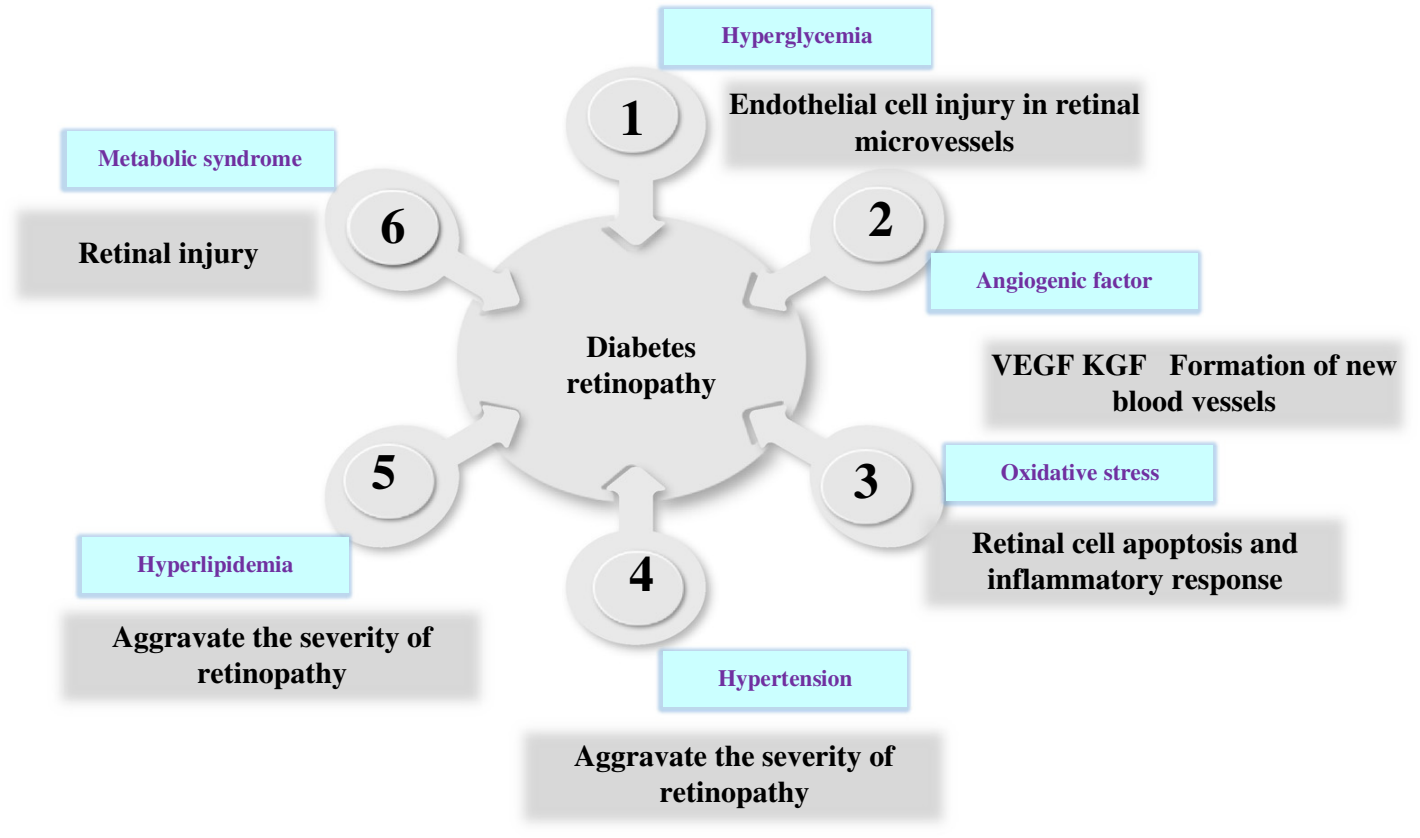
Schematic diagram of the pathogenesis of DR.

There are two types of DR: non-proliferative DR (NPDR) and proliferative DR. NPDR mainly includes microangiopathy and sclerotic lesions, while hyperplastic DR occurs on the basis of NPDR, including neovascularization and vitreous hemorrhage. The pathogenesis of DR is complex, which is related to hyperglycemia, vascular hyperplasia factors, oxidative stress, hypertension, hyperlipidemia, and other factors [13]. The hyperglycemia state of diabetes patients will lead to the damage of endothelial cells in retinal micro-vessels, increase the permeability of capillaries, and then promote the seepage of retina, the formation of edema, vitreous hemorrhage, and other diseases. Hyperglycemia state can also stimulate retinal vascular endothelial cells and retinal pigment epithelial cells to release a variety of vascular proliferation factors, such as vascular endothelial growth factor (VEGF) and keratine epithelial growth factor. These factors can cause capillary neovascularization and the formation of new blood vessels, which can then lead to hemorrhage and retinal edema and other diseases [14]. Hyperglycemia state can also lead to increased oxidative stress in the retina, promote retinal cell apoptosis and inflammatory response, and then accelerate the process of retinopathy. Hypertension and hyperlipidemia are common in patients with diabetes, and hypertension may aggravate the severity and progression of retinopathy. Prieto Del Cura and Gonzalez-Guijarro [15] discussed the clinical predictors of DR progression and found that poor blood glucose control, systemic hypertension, diabetes duration, dyslipidemia, and microalbuminuria were the main risk factors for the development and progression of DR. Increased aortic sclerosis has also been identified as a prognostic marker for DR and peripheral neuropathy. Bu et al. [16] found that patients with DR may be related to hypertension, high body mass index, metabolic abnormalities, and duration of diabetes, and found that aging, other complications, and metabolic syndrome are also related to DR. The pathogenesis of DR is very complex and involves the interaction of many factors. Therefore, in the aspect of prevention and treatment of DR, a comprehensive consideration of many factors should be taken to take effective measures for treatment and management. Figure 1 shows the pathogenesis of DR.

#### 2.1.2 Treatment

Blood sugar control is the most basic method to treat DR. By controlling blood sugar level, the occurrence and progression of retinopathy can be reduced or delayed. Drug treatment mainly includes hormone drugs, photosensitizers, and anti-VEGF drugs, which can reduce retinal edema, control the generation of new blood vessels, and reduce vitreous hemorrhage. Laser therapy is an effective method for the treatment of retinopathy, mainly suitable for early mild DR, including laser photocoagulation and laser photochemistry two ways. Injection therapy is a new treatment method, which can inhibit the generation and growth of new blood vessels and reduce bleeding and edema through injection of anti-VEGF drugs [17]. For severe DR, such as vitreous hemorrhage and retinal detachment, surgical treatment, such as vitrectomy and retinal reduction, is required [18]. For different types and degrees of DR, a variety of treatment methods should be considered comprehensively and individualized treatment programs should be adopted. Meanwhile, attention should also be paid to the prevention and management of the occurrence and progression of diabetes, to fundamentally reduce or avoid the occurrence of retinopathy.

### 2.2 Keratopathy

#### 2.2.1 Pathogenesis

**Figure 2:**
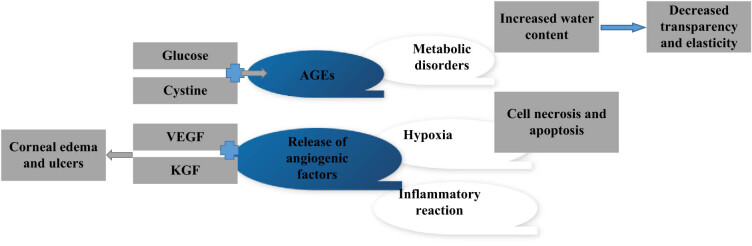
Schematic diagram of pathogenesis of keratopathy.

Diabetes keratopathy is one of the most common OCs in patients with diabetes. The hyperglycemia state of diabetes patients will lead to metabolic disorders of corneal epithelium cells, increase water content of corneal matrix, relaxation of cornea, loss of transparency and elasticity, and occurrence of keratopathy [19]. The state of hyperglycemia promotes the combination of glucose and cystine and other substances to form AGEs, which can accumulate on the cornea, cause damage to the function of corneal epithelium and endothelial cells, and affect the transparency and metabolism of the cornea [20]. The state of hyperglycemia can also lead to hypoxia of retina and cornea, cause cell necrosis and apoptosis, and further aggravate the severity of keratopathy. The state of hyperglycemia can also stimulate corneal endothelial cells and corneal stromal cells to release various vascular hyperplasia factors, cause capillary neovascularity, and then lead to corneal edema, corneal ulcers, and other lesions [21]. The state of hyperglycemia can also lead to the increase of immune inflammatory response in the cornea and promote the process of keratopathy. Zagon et al. [22] found that KCNQ1OT1/miR-214/caspase-1 signaling pathway represents a new mechanism for the progression of endothelial keratopathy in diabetes, and KCNQ1OT1 may be a new therapeutic target. The pathogenesis of diabetes keratopathy is multifaceted, and it is necessary to consider the characteristics of the systemic conditions and eye lesions of diabetes patients and take effective treatment measures. Patients with diabetes should prevent and treat the occurrence of diabetes keratopathy by checking their eye health status regularly, controlling blood sugar and blood pressure. Figure 2 shows the pathogenesis of keratopathy.

#### 2.2.2 Treatment

The main treatment of diabetes keratopathy is to control blood sugar level, which can slow down the process of keratopathy and reduce the risk of developing diabetes keratopathy [23]. Common methods to control blood sugar include diet, exercise, oral medication, and insulin injections. If diabetes patients develop ocular surface xerosis, artificial tear eye drops can be used to lubricate the cornea and relieve symptoms [24]. Some repairing additives can help corneal epithelial cells repair damaged tissue and promote the repair and regeneration of the corneal surface. Anti-inflammatory drugs, such as corneal lubricants or topical corticosteroids, can be used for inflammatory reactions caused by diabetes keratopathy [25]. For vision problems caused by keratopathy, optical treatments such as orthokeratology lenses or corneal transplants can be applied to improve vision. Some implantable medical devices, such as corneal rings, can be used for the treatment of diabetes keratopathy [26]. According to different types and severity of diabetes keratopathy, doctors will choose different treatment methods or combination therapy. At the same time, patients with diabetes should also actively control blood sugar, blood pressure and blood lipid, and check eye conditions regularly to prevent the occurrence of diabetes keratopathy.

### 2.3 Xerophthalmia

#### 2.3.1 Pathogenesis

**Figure 3:**
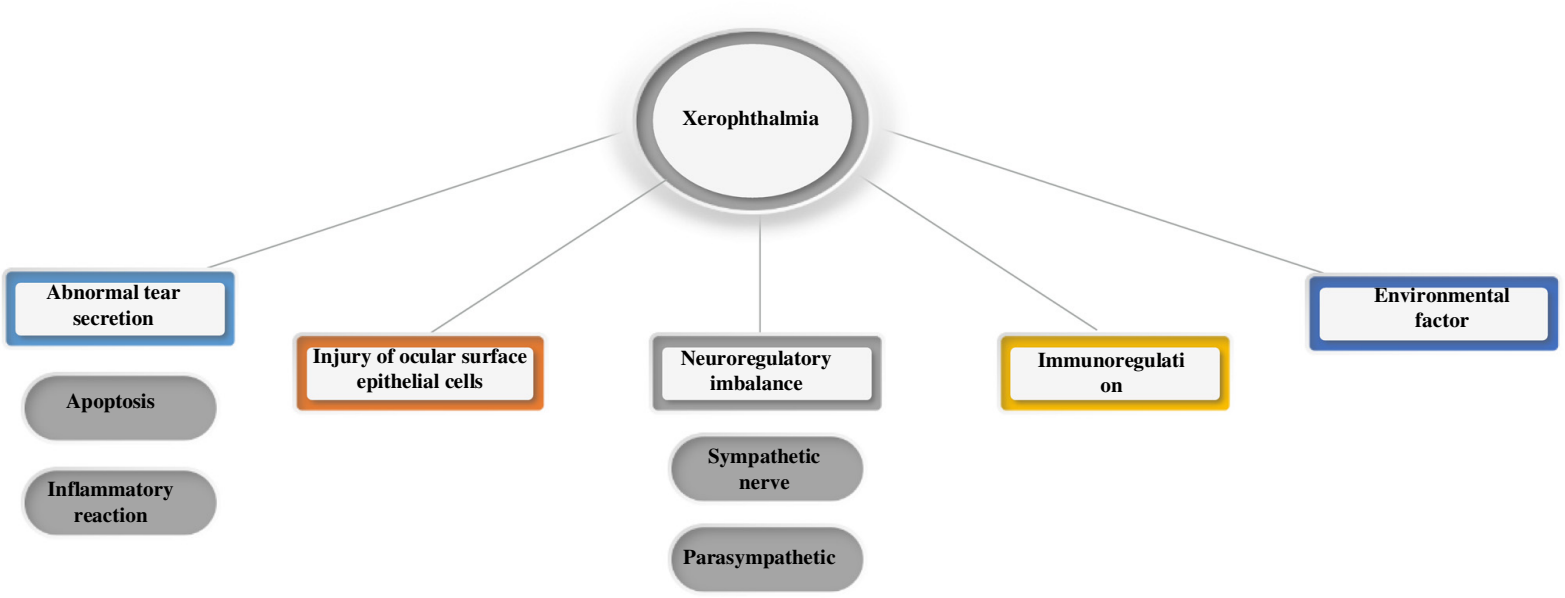
Pathogenesis of xerophthalmia.

Xerophthalmia caused by diabetes is a metabolic disease, which is related to the abnormal stability of tear film, with high incidence and difficult treatment. The tear film is a three-part coating of tears, mucus, and lipids that lubricates and protects the surface of the eye. The amount and quality of tears in patients with xerophthalmia are reduced, and the stability of tear film is decreased, leading to symptoms such as dryness and pain on the ocular surface [27]. Tears are secreted by lacrimal glands, auxiliary lacrimal glands, and other parts. In xerophthalmia patients, tear secretion in these parts is reduced or abnormal, resulting in insufficient lubrication of tears on the ocular surface. Environmental factors, too much time on electronic devices, and reduced blinking can cause tear film to evaporate too quickly, exacerbating eye fatigue and dryness. In patients with xerophthalmia, ocular surface epithelial cells are damaged or destroyed, which can easily lead to apoptosis of corneal epithelial cells and inflammatory reactions, aggravating xerophthalmia symptoms [28]. Studies have found that the imbalance of nerve regulation in xerophthalmia patients may also be one of the pathogeneses. The sympathetic and parasympathetic nerves play an important role in tears secretion and tear film stability, and the imbalance of nerve regulation may lead to reduced tears secretion or instability of tear film [29]. In addition, the immune regulation of patients with xerophthalmia may be disturbed, leading to ocular surface inflammation and autoimmune diseases, which may aggravate xerophthalmia symptoms. The onset of xerophthalmia is also related to other factors, such as drugs, diseases, surgery, and keratopathy. The pathogenesis of xerophthalmia is multifaceted, which requires comprehensive consideration of the systemic conditions and the characteristics of ocular lesions in patients with xerophthalmia, and effective treatment measures should be taken. Figure 3 demonstrates the pathogenesis of xerophthalmia.

#### 2.3.2 Treatment

Patients with xerophthalmia can undergo artificial tear drops, which lubricate the eyes and relieve symptoms of xerophthalmia. Depending on the specific condition of patients, doctor will recommend different types of artificial tears, including eye drops, creams, and more. Patients with xerophthalmia need to avoid working and living in unhealthy environments such as air-conditioned rooms and prolonged use of computers. In dry environments, the use of humidifiers can increase air humidity and help relieve xerophthalmia symptoms [30]. Patients with xerophthalmia can use some medications to relieve symptoms, such as short-term use of antihistamines and non-steroidal anti-inflammatory drugs. In addition, some artificial tears also contain pharmaceutical ingredients that can be therapeutic. Patients with abnormal neuromodulation in xerophthalmia may be treated with neuromodulation drugs to regulate the nervous system and improve symptoms. Some surface repair agents can help corneal epithelial cells repair damaged tissues and promote the repair and regeneration of corneal surface [31]. For vision problems caused by xerophthalmia, optical treatments such as orthokeratology lenses or corneal transplants can be used to improve vision. For some patients with severe xerophthalmia, surgical treatment may be considered. Surgical treatment includes lacrimal gland ostomy and lacrimal gland transplantation. Prasad et al. [32] discussed the effect of integrated Chinese and western medicine in treating diabetes related xerophthalmia, and found that integrated Chinese and western medicine may be effective in treating diabetes xerophthalmia, and inflammatory factors are potential biomarkers to test the therapeutic effect. The treatment of xerophthalmia requires different treatment methods or combination therapy according to the different conditions. Patients with xerophthalmia should actively control environmental factors, maintain good living habits, and undergo regular eye examinations to prevent the deterioration of the condition.

### 2.4 Glaucoma

#### 2.4.1 Pathogenesis

**Figure 4:**
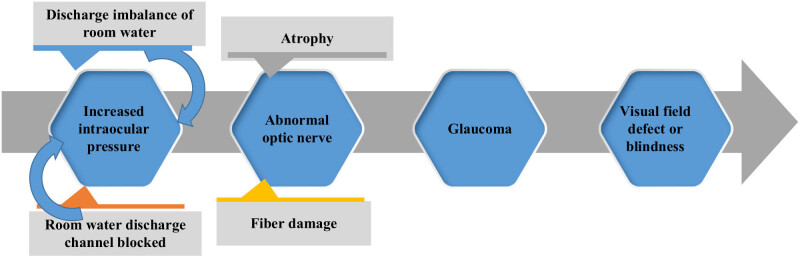
Pathogenesis of glaucoma.

The main characteristic of glaucoma is increased intraocular pressure, which is an important factor in the onset of glaucoma. Increased intraocular pressure may be due to uneven production and discharge of aqueous humor, or may be due to obstruction of aqueous humor discharge channels. Increased pressure in the eye can lead to damage of the optic nerve, which can affect vision. Li et al. [33] found that diabetes may lead to glaucoma optic neuropathy through increasing intraocular pressure or vascular lesions and direct damage to the optic nerve. Long-term high intraocular pressure can lead to atrophy of the optic nerve head and damage to optic nerve fibers, eventually leading to visual field loss or blindness. Glaucoma patients suffered ischemia, hypoxia, and atrophy of optic nerve and retinal blood vessels, leading to optic nerve atrophy and vision loss. In addition, ocular tissues of patients with glaucoma may have inflammatory reactions, resulting in increased intraocular pressure and optic nerve damage [34]. Glaucoma may have a certain genetic predisposition, and some people are at higher risk for the disease. Some gene mutations may lead to glaucoma-related symptoms such as increased intraocular pressure and optic nerve damage [35]. The onset of glaucoma may also be related to a number of other factors, such as age, gender, race, medication, and disease. The pathogenesis of glaucoma is multifaceted. Early detection, early intervention, and effective treatment can reduce the occurrence and development of glaucoma and protect patients’ vision. Figure 4 shows the schematic diagram of the pathogenesis of glaucoma.

#### 2.4.2 Treatment

The main treatment for glaucoma is drug therapy, which is the most common and effective way to control intraocular pressure. Drugs can slow and prevent damage to the optic nerve by reducing pressure in the eye. Drugs commonly used include drugs to reduce intraocular pressure, drugs to promote aqueous humor discharge, drugs to inhibit aqueous humor production, etc. Laser therapy can reduce intraocular pressure by enhancing the channel at the angle of the chamber to promote the discharge of aqueous humor [36]. The commonly used laser treatment methods include three kinds of laser therapy and laser small incision surgery. For some patients with refractory glaucoma, surgical treatment is needed to reduce intraocular pressure. The commonly used surgical treatment methods include filtration surgery and implant surgery [37]. Conservative treatment involves paying attention to lifestyle and keeping your eyes healthy. For example, it should avoid strenuous exercise and lifting heavy objects, get enough sleep, stop smoking, and limit alcohol consumption, and protect your eye health through eye exercises, eye massages, and eye hygiene. Patients with glaucoma should have their eye pressure, vision, and visual field checked regularly and receive timely treatment to prevent the condition from worsening.

### 2.5 Cataract

#### 2.5.1 Pathogenesis

**Figure 5:**
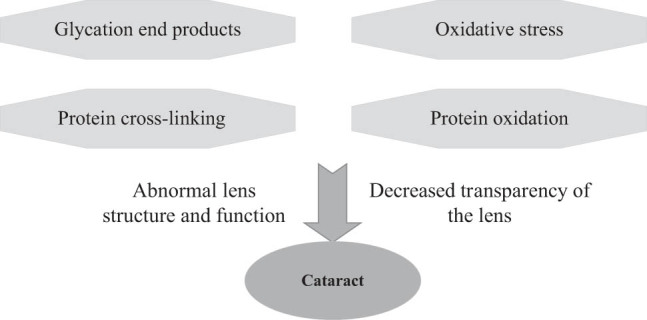
Pathogenesis of cataract.

Cataract is a common eye disease in the elderly. It is a cataract of vision disorders caused by opacity of the lens. Oxidative stress is an important factor in cataract occurrence. Oxidative stress in the body can cause oxidation and damage to proteins in the lens, which can lead to a cataract of decreased transparency. T2DM is a risk factor for cataract occurrence. As the prevalence of T2DM increases, so will the burden of cataract-related vision loss. Dash et al. [38] analyzed specific risk factors of diabetes cataract and found that age and blood sugar control were always correlated with the occurrence of T2DM-induced cataract, but blood pressure, diabetes duration, gender, and aspirin use were not. Lipids and smoking are still possible risk factors. In the state of hyperglycemia, glycation end products will accumulate in the lens, leading to the cross-linking and aggregation of proteins, which will damage the lens structure and function, forming a cataract. Apoptosis of lens cells is also one of the important mechanisms of cataract occurrence. Apoptosis of lens cells leads to a cataract of decreased transparency and aggregation of proteins. Long-term exposure to ultraviolet rays can also lead to oxidation and damage of proteins in the lens, resulting in a cataract of cataract transparency [39]. Long-term use of medications such as steroids also contributes to the cataract. Other factors such as poor nutrition, eye inflammation, and trauma can also cause cataract occurrences. Early detection of interventions and effective treatment can slow the development of cataract and protect patients’ vision. Figure 5 shows the pathogenesis of cataract.

#### 2.5.2 Treatment

Surgery is by far the most common way to treat the cataract, and the most effective. Surgery can be performed to restore the patient’s vision through the replacement of an artificial lens. Common surgical methods include phacoemulsification and grinding. After surgery, it is usually necessary to wear an eye mask and eye drops, pay attention to postoperative care and review. The drug treatments focus on complications such as cataract inflammation and infections before or after surgery. Commonly used drugs include antibiotic eye drops, anti-inflammatory eye drops, and infusion [40]. Conservative treatment includes lifestyle and eye health such as avoiding strenuous exercise and lifting heavy objects, getting enough sleep, stop smoking, and limiting alcohol consumption. In addition, it can prevent cataract by taking the right amount of antioxidants such as vitamins E and C. For some special types of cataracts, such as the cataract of glaucoma and cataract of fundus diseases, different treatment measures should be taken for different causes. Cataract treatments require choosing different treatments, or combinations, depending on the patients’ conditions and causes. Patients should have their eye health checked regularly and receive timely treatment to prevent their condition from worsening.

## 3 Research progress of OCs and function of tears and blepharons

### 3.1 OCs and functions of tears

**Figure 6:**
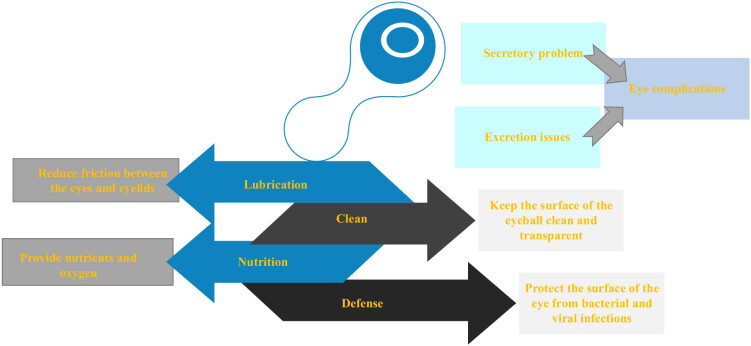
Function of tears and mechanism of eye diseases.

**Figure 7:**
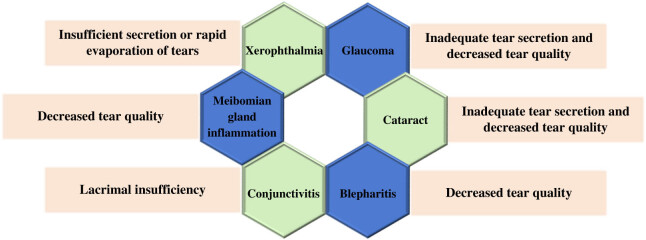
Effect of OCs on tears function.

OCs is closely linked to the tears function. Tear is a protective film on the surface of eyes with functions of lubrication, cleaning, nutrition, and defense, which is one of the important factors in maintaining eye health. It can also provide nutrients and oxygen to protect the surface of eyes from bacterial and viral infections [41]. Eye diseases will affect the secretion and quality of tears, resulting in abnormal tears function. When there are problems with the secretion or excretion of tears, the normal physiological function of the eyes will be affected, resulting in a variety of eye diseases. Xerophthalmia is a disorder caused by insufficient production of tears or rapid evaporation of tears. Lack of tears can cause discomfort such as dry, sore, and itchy eyes. Common features of dry eye syndrome (DES) are abnormal tear film, and abnormalities associated with DES are the absence of tears due to insufficient supply or excessive loss, and abnormal tears composition. DES can disrupt the homeostasis of tear film and its adjacent structures and adversely affect its basic functions (such as supporting ocular surface epithelium and preventing microbial invasion) [42]. Treatments for xerophthalmia include lubricant drops, hot compresses, and eye massages aimed at increasing tears secretion and improving their quality. Glaucoma is a disease of the eye that causes damage to the optic nerve, often accompanied by increased pressure inside the eye. High intraocular pressure can affect the secretion and excretion of tears, resulting in insufficient tears and reduced tear quality. Treatments for glaucoma include lowering the pressure in the eye and lubricant drops to improve the quality of the tears and relieve discomfort. Cataract is a common eye disease in the elderly, which causes opacity of the lens, which affects vision [43]. Cataract patients often have symptoms of insufficient secretion of tears and decreased quality of tears. Cataract treatments include surgery and lubricant drops to improve the quality of tears and protect the surface of the eye. Blepharitis are eye diseases caused by bacterial infections caused by impaired secretion of sebaceous glands at the edges of the eyelids. Blepharitis patients often suffer from decreased quality of tears, resulting in uncomfortable symptoms such as dry and stinging eyes. Treatment for blepharitis includes eye cleaning, antibiotic eye drops, and hot compresses to improve the quality of tears and reduce inflammation [44]. Conjunctivitis can also cause discomfort such as reduced tears, red eyes, itching, and foreign body sensation. Blepharons adenitis can lead to the deterioration of tears quality, thus causing eye discomfort, blurred vision, and other symptoms [45]. Function of tears is very important to the health of the eyes, and various eye diseases are closely related to tears function. Therefore, to maintain good living habits, pay attention to eye hygiene, reasonable use of eyes, etc., are conducive to the maintenance of tears function and eye health. Figure 6 shows the function of tears and the pathogenesis of eye diseases, and Figure 7 demonstrates the effect of OCs on the function of tears.

The change of tear secretion in diabetes patients is related to multiple peripheral neuropathies in diabetes patients [46,47], abnormal glucose metabolism [48], neurotransmitters [49,50], and extracellular matrix components [51]. In diabetes, the blood sugar increases, the content of inositol in nerve tissue decreases, the metabolism of phosphatidylinositol decreases, and the activity of protein kinase C decreases, which affects the phosphorylation of Na^+^–K^+^ ATPase, resulting in the decrease of activity, the increase of intracellular Na^+^, the decrease of transmembrane Na^+^ concentration gradient, the slowing of nerve conduction speed, the decline of corneal sensation, and the reduction of tear secretion. Herber et al. found that the tear protein composition of DM patients is different from that of normal individuals [52]. Kalló et al. showed that diabetes patients are different from normal people in terms of tear protein content and saturation, and also different from ordinary dry eye patients. The change of tear protein in diabetes patients is related to the course of diabetes, and the change is obvious in patients with long course of disease [53]. Nokhoijav et al. found that Apo (a-I) in tears of T2DM patients was significantly increased and closely related to the severity of DR, but no expression of Apo (a-I)-related genes was detected [54]. Kuo et al. found that the distribution of tear film lipid layer (TFLL) in DM patients was uneven, tear film rupture time and corneal sensation were decreased, suggesting that the function of tear lipid layer in diabetes patients was reduced and related to diabetes corneal epithelial lesions [55].

### 3.2 OCs and function of blepharons

**Figure 8:**
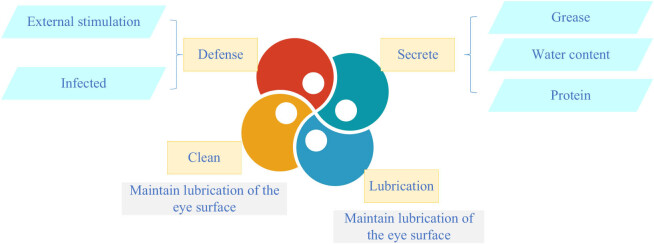
Functional diagram of blepharons.

Blepharons are secretory organs located on the inner side of the eyelid, which are part of the eyelid and consist of glands, glandular ducts, and glandular ducts that act as a protective barrier to the eye. Its main function is to secrete oil, water, protein, and other substances, maintain the lubrication and protection of the eye surface. Blepharons not only keep tears stable and clean, but also protect the ocular surface area from external irritation and infection. Studies have shown that the TFLL stabilizes the air/tears surface of the human eye. Blepharons glandular dysfunction (MGD) can lead to quantitative and qualitative changes in TFLL main components (>93%). The oily secretion of blepharons lipid is the main cause of DES, and up to 86% of DES patients will show signs of MGD [56]. OCs are closely related to the function of blepharons, and the malfunction of blepharons will lead to a series of eye diseases. Blepharons adenitis is one of the main causes of blepharons dysfunction, which can lead to a decrease in the quality and quantity of blepharons secretions, thus affecting eye lubrication and protection. Symptoms of blepharons adenitis include dry eyes, eye pain, foreign body sensation, blurred vision, and so on. The blepharons gland secretes blepharons, which produce the lipid layer of the tear film, thus preventing excessive evaporation of tears. MGD is the primary cause of evaporative xerophthalmia, which is more common than dehydrated xerophthalmia. Non-invasive blepharons photography relies on infrared light and infrared sensitive cameras to reveal the morphology of blepharon glands in the upper and lower eyelids, while tears interferometry can be used for qualitative and quantitative evaluation of the lipid layer of the tear film [57]. Xerophthalmia is an eye disease caused by insufficient eye lubrication, and blepharons dysfunction is one of the main causes of xerophthalmia. Blepharons secrete oils that lubricate the tears and form a protective film that reduces their evaporation. If the blepharons do not produce enough oil, they can cause tears to evaporate too quickly, which can cause symptoms such as dry eyes, discomfort, and blurred vision. Blepharons disorder refers to the disorder of secretion and excretion of blepharons secretions, resulting in insufficient oil secretion or oil quality decline. Blepharons disorder will make the eye surface not smooth, causing dry eyes, pain, blurred vision, and other symptoms [58]. Blepharons is very important for eye health. To protect blepharons function, it is necessary to pay attention to eye hygiene, avoid eye infection and irritation, have regular eye examinations, and timely detect and treat blepharons function abnormalities and other eye diseases. In addition, some eye care measures, such as eye massage, hot compress, nutritional supplement, can also be performed to help restore and maintain blepharons functional health. Figure 8 illustrates the functional of blepharons.

**Figure 9:**
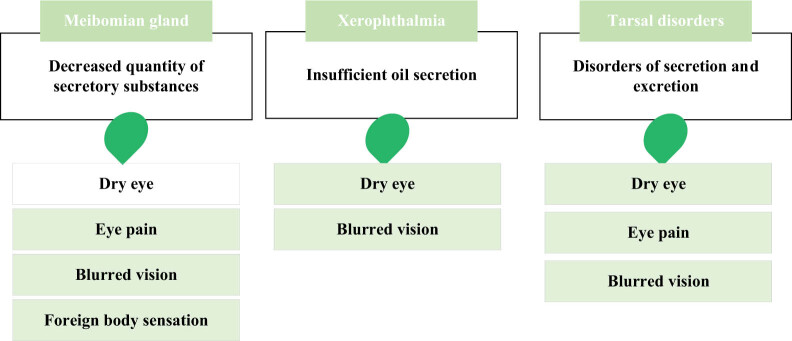
Effect of eyelid function on OCs.

At present, few studies have focused on the influence of diabetes on the morphology and function of meibomian gland [59]. A large-scale epidemiological study found that diabetes is a risk factor for asymptomatic meibomian gland dysfunction (MGD) [60]. MGD is the most common cause of evaporative dry eye, which can alter the quality and quantity of lipids in the secretion of the meibomian gland [61,62]. Previous studies have proved that T2DM patients have more severe MGD than non-diabetes patients [63]. Fan et al. reported that compared with the healthy control group in their study, diabetes patients showed significantly worse meibomian gland changes, including higher meibomian gland abscission rate, lower number of expressible glands, and higher meibomian margin abnormality score [64]. At the same time, studies have pointed out that the loss rate of upper and lower meibomian glands in T2DM patients is significantly higher than that in non-diabetes patients [65]. Figure 9 demonstrates the impact of eyelid function on OCs.

## 4 Application of DL in T2DM and OCs

DL is an artificial intelligence (AI) technology that enables the diagnosis and treatment of eye diseases by automatically extracting features and building models by learning and analyzing large amounts of data. In recent years, DL has been widely used in the field of eye diseases. DL is a special type of machine learning that can achieve relatively more efficient learning outcomes. It is a method of classifying and regressing various types of data by training multi-layer artificial neural network structures. DL is a type of learning that uses deep neural networks to process feature representation. After data input, the input data is abstracted into features layer by layer through a multi-layer network, and finally the features are converted into task targets. The feature abstraction process is entirely completed by the system network itself, without adding any manual operations. DL is a kind of machine learning technology based on neural network, which can automatically extract, learn, and understand features in data, and carry out tasks such as classification and recognition. In the field of eye diseases, DL can realize automatic diagnosis and classification of eye diseases through the analysis and processing of eye images and data, improving the accuracy and efficiency of diagnosis. Through DL algorithm, fundus images can be automatically analyzed, which can realize the accurate diagnosis of DR, macular degeneration, and other diseases. The specific implementation of DL is to construct a neural network to automatically discover the inherent laws and potential representations in training data to serve the ultimate goal [66]. At present, multiple DL algorithms have been applied to T2DM and OCs. The classification advantage of DL is mainly that it can automatically adjust and update the network weight feature parameters after the image is input to the convolutional neural network, directly achieving an “end-to-end” classification mode, eliminating the need for manual feature extraction [67]. The U-Net network [68] performs image segmentation through data augmentation, preserving more detailed feature information through rich skip connections. The network fuses deep and shallow features of the same scale through skip connections to obtain more detailed multi-scale feature information. CE Net network [69] proposes a context feature extraction module based on the U-Net network framework, which has achieved good results in the segmentation of retinal blood vessels and optic discs in the fundus. At present, it has been used in the research related to the classification of diabetes retinopathy. Under the joint action of convolution, pooling, nonlinear activation, and other operations, the convolution neural network extracts the feature expression of the original data from the input layer, and then carries out layer by layer abstract operations [70]. The convolution neural network can identify low specificity features such as bleeding, microaneurysms, and so on. Based on this, it classifies the images of diabetes retinopathy at five levels [71].

**Figure 10:**
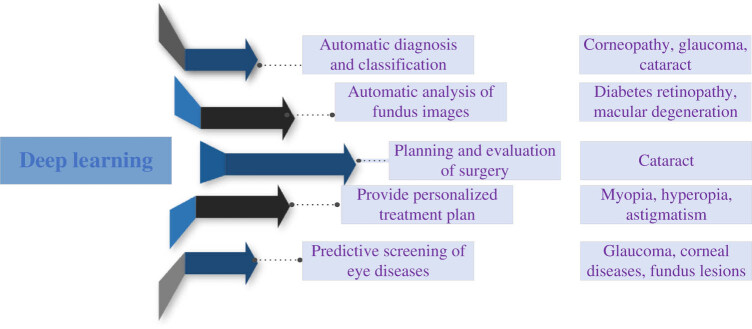
Application of DL in T2DM and OCs.

Tan et al. [72] found that DL algorithm can be an effective tool for risk stratification and screening of myopic macular degeneration and high myopia in some myopic people around the world. DL can also be applied to the diagnosis and treatment of eye diseases such as keratopathy, glaucoma, and cataract. Li et al. [73] developed a clinically feasible DL system for prediction of onset and progression of glaucoma based on color fundus photographs and stratified risk prediction, as well as clinical validation of the performance of an external population cohort. They found that AI model has high sensitivity and specificity to predict the incidence and progression of glaucoma in the future, which proves the feasibility of DL algorithm in the early detection and prediction of glaucoma progression. DL technology can automatically extract lesion features through the study and analysis of many retinal images to realize automatic diagnosis of retinopathy, including DR and macular degeneration. Zekavat et al. [74] used CNN to segment retinal micro-vessels, and calculated blood vessel density and fractal dimension as a measure of the complexity of blood vessel branches. Low retinal vascular fractal dimensions and density were found to be significantly associated with higher risk of event mortality, hypertension, congestive heart failure, renal failure, T2DM, sleep apnea, anemia, and multiple ocular diseases, as well as corresponding quantitative characteristics. DL technology can also automatically extract ocular structures and surgical risk features by learning and analyzing eye images, and realize planning and evaluation of cataract operations, including preoperative ocular morphology analysis and surgical risk assessment. In addition, DL technology can automatically identify different types of visual impairments, including myopia, farsightedness, astigmatism, etc., through the learning and analysis of vision test data, and provide personalized treatment plans. DL technology can automatically identify disease risk factors and lesion characteristics through the study and analysis of a large number of ocular disease data, so as to realize the prediction and screening of ocular diseases, including glaucoma, corneal diseases, and fundus lesions. Tsiknakis et al. [75] analyzed various steps of DL method in the DR detection pipeline based on fundus images, and proposed an AI-driven method for detecting and classifying DR on fundus retinal images, thus improving the detection and diagnosis efficiency. DL technology has a wide application prospect in the field of ophthalmology and is expected to provide more accurate and efficient solutions for the diagnosis, treatment, and prevention of eye diseases [76]. At present, the research and application of DL in the field of eye diseases is still in its infancy and needs further research and exploration. The continuous development and application of DL technology will bring more possibilities for the early diagnosis and treatment of eye diseases. Figure 10 shows the application of DL in T2DM and OCs.

Although the DL algorithm has potential application prospects in the research of diabetes-related ophthalmopathy, the structural features of diabetes retinopathy images are not obvious, and the overall signal-to-noise ratio is not high, which leads to many network models being unable to effectively extract features during training. Hence, it needs further research. In addition, in the application of DL in diabetes-related eye diseases, challenges such as data demand, opacity, generalization ability, data deviation, privacy issues, and sample imbalance need to be overcome to ensure its effectiveness and reliability in clinical practice.

## 5 Conclusion

T2DM is difficult to treat, with many complications and complex clinical symptoms, which seriously threaten the normal life of patients. Patients with T2DM and OCs without timely treatment will worsen the disease and increase the rate of blindness in patients. Tears and blepharons, as two important secretions on the surface of eyes, have the function of protecting the health of the surface of eyes. The occurrence of OC may be affected by abnormal function of tears and blepharons. This work reviewed the main OCs of T2DM and its pathogenesis and treatment, analyzed the correlation between OCs and function of tears and blepharons, and explored the application of DL in T2DM and OCs. It aimed to give a theoretical basis for the prevention, control, and treatment of patients with T2DM and OCs.
